# Optically‐Coupled X‐Ray Computed Laminography System for High‐Speed Inspection of Lithium‐Ion Batteries

**DOI:** 10.1002/advs.202517158

**Published:** 2025-11-21

**Authors:** Jaeyoung Im, Jun Heo, Seunguk Cheon, Jichan Kim, Sung Oh Cho

**Affiliations:** ^1^ Department of Nuclear and Quantum Engineering Korea Advanced Institute of Science and Technology (KAIST) 291 Daehak‐ro, Yuseong‐gu Daejeon 34141 Republic of Korea

**Keywords:** high‐speed inspection, lithium‐ion battery, optical magnification, X‐ray computed laminography

## Abstract

Conventional X‐ray computed tomography systems for microstructure inspection typically employ a geometrically magnified imaging setup, requiring a micro‐focus X‐ray source to mitigate image blurring caused by a finite focal spot size. However, due to its low output power, the micro‐focus X‐ray source results in prolonged image acquisition times. This study presents a novel optically‐coupled X‐ray computed laminography system for high‐speed inspection. In the proposed system, a scintillator is placed in contact with an object, effectively minimizing image blurring despite the use of an X‐ray source with a large focal spot. Consequently, a high‐power X‐ray tube can be employed without compromising spatial resolution, leading to a significant reduction in scan time. Modulation transfer function analysis confirms that a spatial resolution of 38 µm is achieved using a high‐power (400 W) X‐ray tube with a 400 µm focal spot. In addition, high‐quality 3D imaging of lithium‐ion batteries (LIBs) is demonstrated with a scan time as low as 2 s and a contrast‐to‐noise ratio exceeding 3. By overcoming the trade‐off between the spatial resolution and the scan time in X‐ray imaging, the proposed system enables high‐throughput in‐line inspection of mass‐produced LIBs.

## Graphical abstract

This study presents an optically coupled X‐ray computed laminography system for high‐speed inspection of lithium‐ion batteries. A scintillator serving as a virtual detector decouples spatial resolution from the X‐ray focal spot size, enabling defect detection even with large‐spot, high‐power X‐ray tubes. This design achieves rapid 3D imaging and in‐line identification of fine internal defects within seconds.

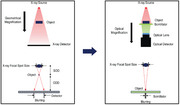

## 1 Introduction

The demand for lithium‐ion batteries (LIBs) has rapidly increased due to the growth of electric vehicles and large‐scale energy storage systems.^[^ ^1^ ^]^ As LIBs play an increasingly critical role in modern technologies, ensuring their reliability and safety through rigorous quality control has become paramount. Throughout the complex manufacturing processes of LIBs, various internal defects can arise—such as electrode misalignment, overhang, burrs on tabs, deflected or cracked electrodes, housing deformation, and uneven winding.^[^ ^2^ ^]^ These internal imperfections can compromise the integrity of the cell under mechanical, thermal, or electrical stress, potentially leading to internal short circuits, gas evolution, reduced cycle life, or even thermal runaway.^[^ ^3^, ^4^ ^]^ For instance, electrode misalignment can induce internal short circuits and accelerate cell degradation due to localized current density.^[^ ^5^ ^]^ If left undetected, such failures may escalate during operation, resulting in serious safety hazards and economic losses.^[^ ^6^ ^]^ Therefore, the early detection and precise localization of internal defects within LIBs are essential to secure battery performance, ensure user safety, and enable quality assurance in high‐throughput, automated manufacturing environments.^[^ ^7^, ^8^ ^]^

Numerous defects arise during the final assembly process and remain undetected within sealed LIBs, highlighting the importance of comprehensive inspection of assembled cells.^[^ ^3^, ^9^ ^]^ These internal defects cannot be identified or located using electrical or optical inspection techniques. Electrical tests determine cell defects by measuring impedance, but they cannot localize or distinguish internal mechanical defects. Similarly, optical inspections can detect surface defects, but they cannot visualize the interior of cells due to the sealed housing.^[^ ^3^, ^10^ ^]^ Instead, nondestructive evaluation methods capable of probing internal structures, such as ultrasonic testing and infrared thermography, have been explored for LIBs inspection.^[^ ^11^, ^12^ ^]^ However, these techniques are limited in their ability to resolve fine internal structures due to low penetration depth or insufficient spatial resolution for detecting micro‐scale defects within sealed cells.^[^ ^13^, ^14^ ^]^ Among these techniques, X‐ray computed tomography (CT) has emerged as a powerful tool for acquiring volumetric images of LIBs, enabling internal defect detection without disassembly.^[^ ^15^ ^]^

Currently, X‐ray CT systems utilize geometric magnification to observe fine internal structures within batteries. This magnification approach relies on the ratio of the source‐to‐object distance (SOD), and the object‐to‐detector distance (ODD). In detail, the magnification factor increases as the ODD‐to‐SOD ratio increases.^[^ ^16^, ^17^ ^]^ However, higher geometric magnification inevitably leads to increased blurring size due to the finite size of the X‐ray focal spot, which scales proportionally with ODD.^[^ ^18^ ^]^ To minimize blurring, micro‐focus X‐ray tubes, which concentrate the electron beam down to several micrometers, are often used. Unfortunately, micro‐focus X‐ray tubes are fundamentally limited in the achievable electron beam power, as most of the beam energy is converted to heat, compromising the durability of the target anode due to thermal stress.^[^ ^19^, ^20^, ^21^, ^22^ ^]^ Consequently, the low X‐ray flux produced by limited electron beam power demands prolonged exposure times to obtain reliable projection images. Acquiring a 3D image typically requires hundreds of projection images, further extending the total inspection time. Moreover, as the focal spot size decreases, thermal stress of the target anode can cause focus drift, shifting the position of the focal spot.^[^ ^23^ ^]^ Therefore, source stabilization time is required to suppress focus drift and the resulting geometric errors during the 3D reconstruction.^[^ ^24^, ^25^ ^]^ As a result, achieving high‐resolution X‐ray imaging traditionally requires high geometric magnification, which in turn necessitates the use of small focal spot X‐ray sources. Thus, the higher the desired spatial resolution, the longer the inspection time—a fundamental trade‐off in conventional X‐ray systems. This trade‐off is particularly problematic in LIBs inspection, where a spatial resolution below 50 µm is typically required, inevitably resulting in extended scan durations. Given that typical production lines manufacture more than 10 cells per minute, it becomes impractical to inspect every cell individually while maintaining the required delivery time. Therefore, current manufacturing lines rely on sampling or intermittent inspections instead of full in‐line inspection. This limitation raises the risk that defective cells with internal anomalies may pass undetected through quality control and ultimately be delivered to customers, potentially resulting in product failures or safety issues in the field.^[^ ^8^, ^10^, ^26^, ^27^ ^]^

To overcome these limitations, we have developed an optically‐coupled X‐ray computed laminography (CL) system that replaces geometric magnification with pure optical magnification via a scintillator–optical system assembly. CL is a 3D imaging technique in which the rotation axis is inclined with respect to the X‐ray beam, making it particularly suitable for planar samples. In recent years, CL has gained increasing attention for high‐resolution imaging and nondestructive inspection.^[^ ^28^, ^29^ ^]^ Building upon these advances, the proposed configuration further decouples spatial resolution from the X‐ray focal spot size by placing the scintillator in direct contact with the object while employing optical magnification. This design enables the use of high‐power X‐ray tubes with large focal spots without compromising image quality, thereby significantly reducing exposure time and eliminating the need for source stabilization intervals. Our objective is twofold: 1) to demonstrate, through modulation transfer function (MTF) analysis, that the proposed CL system maintains the desired spatial resolution under a large focal spot size, and 2) to validate its applicability to in‐line inspection of LIBs by acquiring high‐quality 3D images of pouch‐type cells within short scan times. Pouch‐type cells were chosen as the representative format because their structural susceptibility to external shocks makes internal defects particularly critical.^[^ ^30^ ^]^ Achieving these goals will establish a foundation for in‐line high‐throughput inspection of LIBs in fully‐automated battery manufacturing environments and is expected to bring a new innovation to the design of X‐ray imaging systems.

## 2 Design of the Optically‐Coupled X‐Ray CL System

This section presents the design of the proposed system. It begins by explaining how the optical‐coupling configuration decouples spatial resolution from the X‐ray focal spot size, then analyzes the key factors governing spatial resolution, and finally describes the development of the complete optically‐coupled X‐ray CL system.

### 2.1 Decoupling of X‐Ray Focal Spot Size and Spatial Resolution

**Figure 1:**
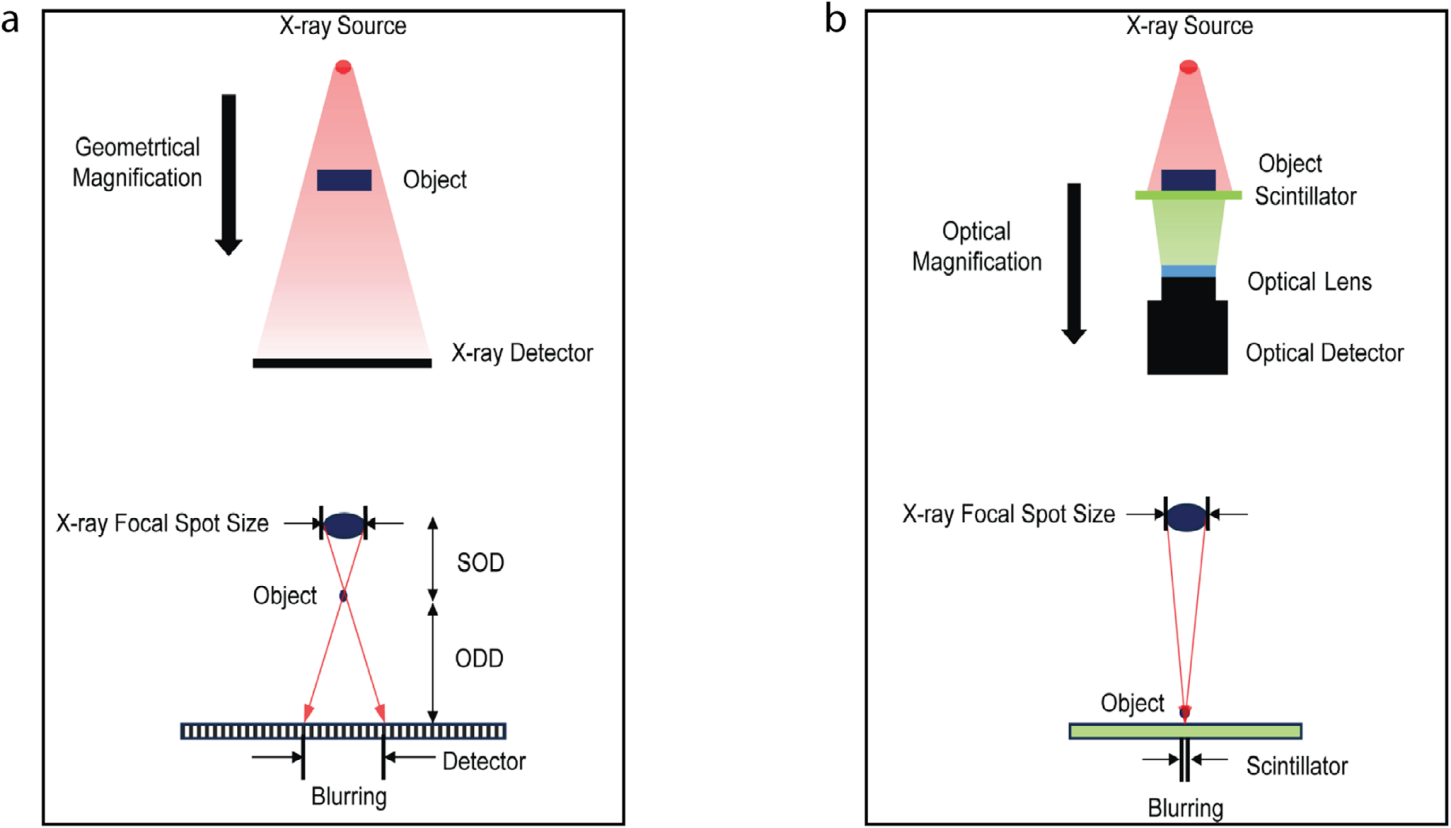
a) Conventional geometric magnification layout and blurring caused by the finite focal spot size of the X‐ray source. b) Proposed optical magnification layout with blurring suppressed by direct contact with the scintillator.

In conventional X‐ray imaging, as illustrated in **Figure** **1**a, geometric magnification is employed to enlarge internal structures of objects. In this geometric magnification method, the magnification factor is determined by the values of SOD and ODD:^[^ ^31^ ^]^

(1) $$\mathrm{Magnification},\;M=\frac{R_{1}+R_{2}}{R_{1}}$$

where *R*_1_ is the SOD and *R*_2_ is the ODD.

The blurring caused by the finite focal spot size of the X‐ray source is expressed as:

(2) $$\mathrm{Blurring},\;B=\frac{S\;R_{2}}{R_{1}}$$

where S is the focal spot size of the X‐ray source. As magnification increases, *R*_2_‐to‐*R*_1_ ratio increases, resulting in greater blurring.^[^ ^32^ ^]^ In conventional X‐ray imaging systems, *R*_1_ and *R*_2_ are fixed values determined by internal spatial constraints for geometric magnification. If the X‐ray focal spot size is large, the blurring becomes more pronounced, as illustrated in Figure 1a, degrading the spatial resolution. To minimize this blurring, micro‐focus X‐ray tubes with small focal spot sizes are typically required.

In this study, as illustrated in Figure 1b, only optical magnification is used to acquire X‐ray images. The scintillator is used to convert transmitted X‐rays into visible light, and the resulting image is projected onto an optical detector through an optical lens system. By positioning the scintillator in direct contact with the object under inspection, *R*_2_ is made significantly smaller than *R*_1_, allowing the geometric magnification factor (*M*) to approach unity.

(3) $$M=1+\frac{R_{2}}{R_{1}}\;\approx 1\;\left(\mathrm{when}\;R_{2}\ll R_{1}\right)$$

Expressing the blurring from Equation 2 with respect to *M*:

(4) $$B=S\;\left(M-1\right)$$

As *M* approaches 1, blurring approaches zero regardless of the focal spot size S of the X‐ray source. Thus, the proposed X‐ray imaging system uses only optical magnification, reducing *R*_2_ instead of decreasing the focal spot size, effectively decoupling focal spot size from blurring. This enables maintaining spatial resolution even with high‐power X‐ray tubes having large focal spot sizes.

### 2.2 Spatial Resolution of the Optically‐Coupled X‐Ray Imaging System

**Figure 2:**
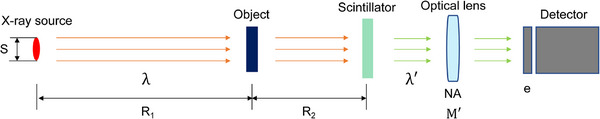
Major parameters influencing spatial resolution in the proposed imaging system.

Beyond suppressing source‐induced blur, the spatial resolution is governed by additional factors summarized in **Figure** **2**. Key resolution‐limiting factors include Fresnel diffraction Equation 5, the focal spot size of the X‐ray source Equation 6, the optical diffraction limit Equation 7, the mismatch between the scintillator thickness and the depth of focus (DoF) of the optical system Equation 8, and the effective pixel size of the detector Equation 10.^[^ ^33^ ^]^

S: Focal spot size of the X‐ray source, λ: X‐ray wavelength, λ′: Scintillator emission wavelength, *R*_1_: SOD, *R*_2_: ODD, NA: Numerical aperture of the optical lens, M′: Optical magnification, e: Pixel size of the detector

Fresnel diffraction occurs when X‐rays pass through an object and propagate to the scintillator, imposing limits on spatial resolution. The spatial resolution limit due to Fresnel diffraction is given by:^[^ ^34^ ^]^

(5) $$R_{\mathrm{Fre}}={\left(R^{\prime }\lambda \right)}^{1/2}$$

where *R*′  =  *R*_2_/*M* is the effective propagation distance. *M* is the geometric magnification.

The blurring caused by the finite focal spot size of the X‐ray source is referred to as the source point spread function (PSF), which is typically modeled as a Gaussian function. In this case, the blur size is equivalent to the full width at half maximum (FWHM) and can be expressed as shown in Equation 2.^[^ ^35^ ^]^ Since the standard deviation (σ) of Gaussian blurring serves as a direct indicator of spatial resolution degradation—and is equal to the FWHM divided by 2.355 in a Gaussian distribution—the resulting spatial resolution limited by the focal spot size is:^[^ ^36^ ^]^

(6) $$R_{\mathrm{source}}=\frac{S}{2.355}\frac{R_{2}}{R_{1}}$$

The spatial resolution degradation due to the diffraction limit of the optical system is:^[^ ^37^ ^]^

(7) $$R_{\mathrm{obj}}=\frac{0.61\;\lambda^{\prime }}{NA}$$

If the position where visible light is emitted from the scintillator lies outside the DoF of the optical system, spatial resolution degradation occurs. If the scintillator thickness exceeds the DoF, the resulting spatial resolution degradation is:

(8) $$R_{\mathrm{scint}}=\frac{\left(\Delta Z-DoF\right)\;NA}{2.355\;n_{\mathrm{scint}}}$$

Equation 8 is applicable only when the scintillator is a single crystal or a structured type. Scintillators made from powder typically exhibit greater degradation in spatial resolution than predicted by Equation 8 due to the scattering characteristics of the powder.^[^ ^38^ ^]^ The derivation is detailed in Figure S1 (Supporting Information).

The DoF can be calculated as:^[^ ^39^ ^]^

(9) $$DoF=\frac{n\;\lambda^{\prime }}{NA}+\frac{n\;e}{M^{\prime }NA}$$

The effective pixel size determined by the optical system also impacts the spatial resolution. Based on the Nyquist–Shannon sampling criterion, the camera resolution is:

(10) $$R_{\mathrm{cam}}=\frac{2\;e}{M^{\prime }}$$

The X‐ray imaging system was designed with careful consideration of these resolution‐limiting factors. Guided by these principles, we developed the optically‐coupled X‐ray CL system, as described in the following section.

### 2.3 Development of the Optically‐Coupled X‐Ray CL System

In conventional CT geometry, where the rotation axis of the object is parallel to the detector, the distance *R*_2_ cannot be reduced due to the object's rotation radius, making it difficult to place the detector close to the object. However, by using the CL geometry, where the rotation axis is not parallel to the detector, it becomes possible to reduce *R*_2_ below the rotation radius. In this configuration, the scintillator acts as a virtual detector, enabling the reduction of *R*_2_.

**Figure 3:**
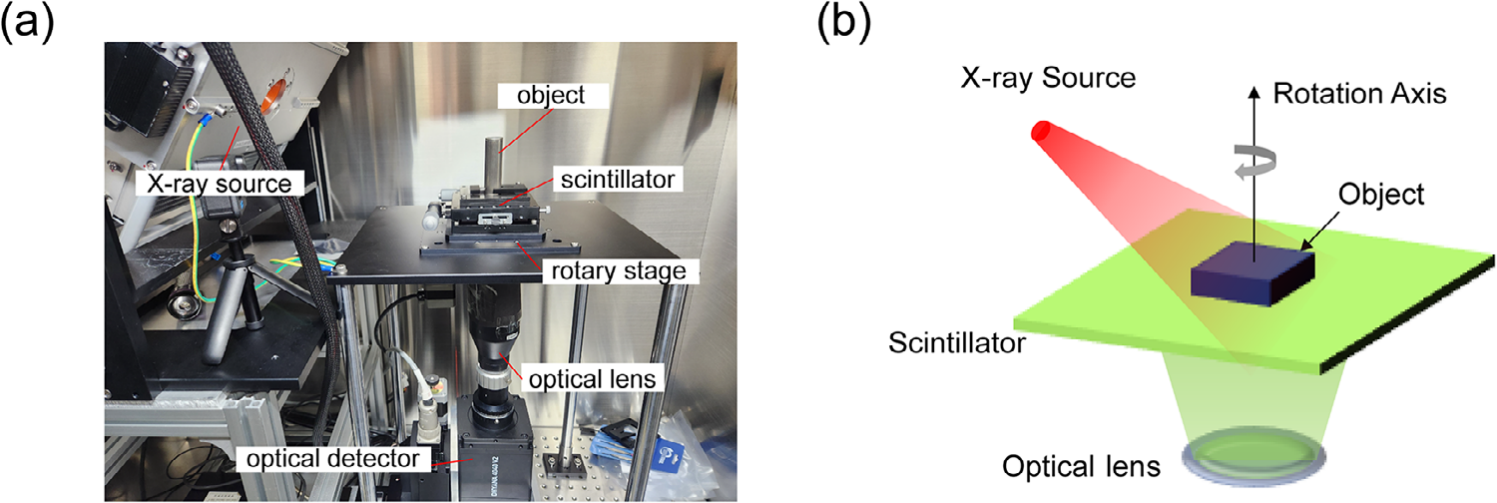
a) Photograph of the developed optically‐coupled X‐ray CL system. b) Schematic diagram illustrating the geometry of 2D projection acquisition in the system.

**Figure** **3**a shows a photograph of the constructed optically‐coupled X‐ray CL system. **Table** **1** summarizes the main specifications of the components comprising the developed X‐ray CL system.

**Table 1:** Specifications of components in the X‐ray CL system.

X‐Ray source	
tube voltage range	80–160 kVp
maximum power	400 W
focal spot size	400 µm

Scintillator	
material	CsI(Tl)
thickness	150 µm
peak emission wavelength	550 nm

Optical lens	
magnification	0.7x
NA	0.087

Optical detector	
pixel size	18 µm
quantum efficiency	75% @ 550 nm

A high‐power X‐ray source (VJ X‐ray, USA) with a 400 µm focal spot was implemented by leveraging the suppression of blurring induced by the focal spot size. The object under inspection was fixed using an acetal holder, and it was positioned in direct contact with the scintillator. A 50 mm × 50 mm × 0.15 mm CsI(Tl) (Hamamatsu photonics, Japan) was used as a scintillator. CsI(Tl) has a columnar structure, minimizing internal scattering due to the waveguide effect, and achieving high light yield. CsI(Tl) has an emission peak wavelength at 550 nm, closely matching the peak quantum efficiency (75%) of the employed sCMOS detector (Tucsen, China). The scintillator thickness was designed to be smaller than the optical DoF. To achieve an effective pixel size of ≈26 µm, an optical lens with 0.7x magnification and a detector with 18 µm pixel size were used.

To ensure accurate alignment of the center of rotation (CoR) during scanning, a manual xy translation stage was mounted on the rotary stage. To align the detector center with the CoR, the *x‐y* axes of the *xyz* stage mounted with the optical detector were adjusted. The *z*‐axis of the *xyz* stage was adjusted to position the scintillator at the working distance of the optical lens, ensuring proper focus.

Figure 3b presents the geometric configuration for X‐ray 2D projection acquisition. The source position is calculated relative to the scintillator center, and distances *R*_1_ and *R*_2_ are defined based on the mid‐plane of the 3D region of interest (RoI) within the object; specifically, *R*_1_ represents the distance from the X‐ray source to the center point of the mid‐plane, and *R*_2_ represents the distance from that center point to the detector. The measured values of *R*_1_ and *R*_2_ are 300.11 mm and 8.34 mm, respectively.

The theoretically calculated spatial resolution components of the developed X‐ray CL system are as follows. The scintillator‐related blurring (*R*_scint_) is not considered, as the scintillator thickness is smaller than the DoF of the optical system, which is 380 µm. The Fresnel blurring term (*R*_Fre_), calculated at an X‐ray energy of 40 keV, is 0.74 µm. The object‐side optical blurring (*R*_obj_) is 3.86 µm, the pixel resolution is limited by the camera pixel size and magnification (*R*_cam_) is 51.43 µm, and the geometric unsharpness due to the X‐ray focal spot (*R*_source_) is 4.72 µm.

## 3 Experiments and Results

### 3.1 Spatial Resolution Measurement of the Developed Imaging System

**Figure 4:**
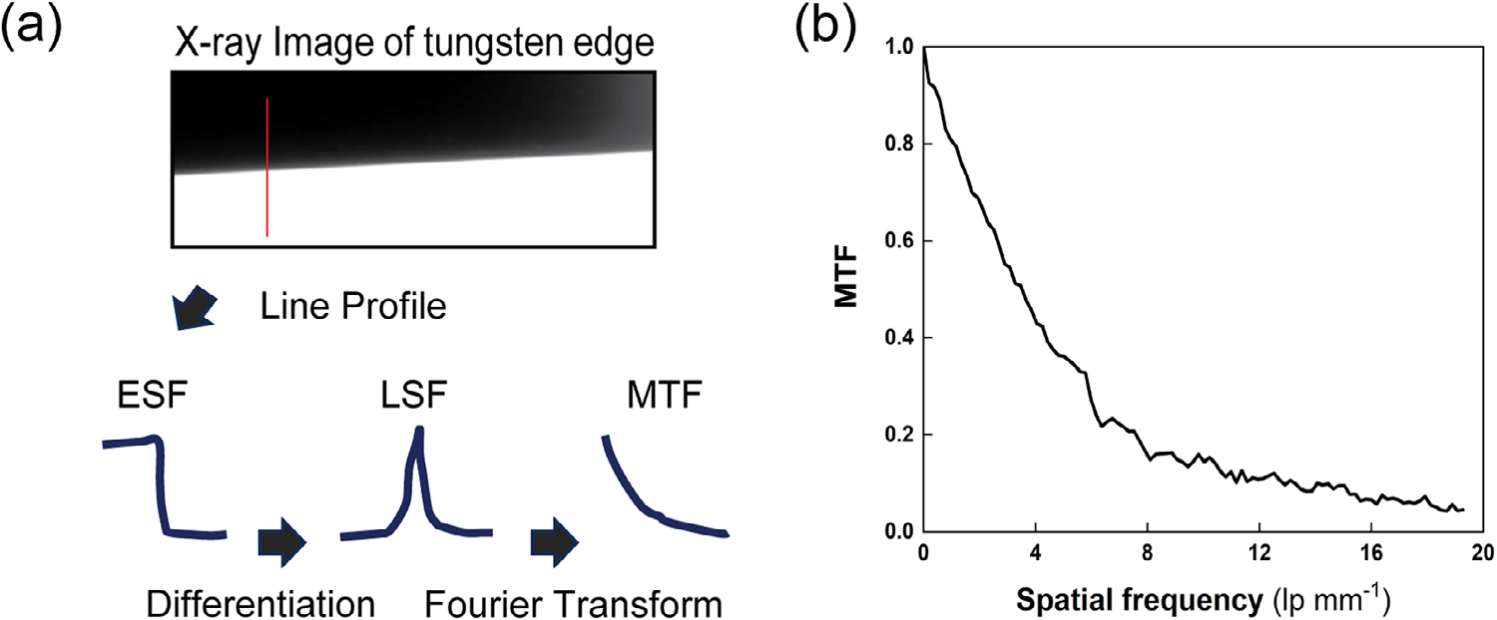
a) MTF measurement procedure. b) Measured MTF graph.

To determine the spatial resolution of the developed X‐ray CL system, the MTF was measured using the slanted‐edge method, a widely accepted standard for evaluating spatial resolution.^[^ ^40^, ^41^ ^]^ **Figure** **4**a illustrates the measurement procedure. A 0.7 mm‐thick tungsten plate with a purity of 99.97% was tilted by ≈2.4° to form a slanted edge, minimizing aliasing.^[^ ^42^ ^]^ A projection image of the tungsten block was acquired at an X‐ray tube voltage of 80 kVp and a current of 1 mA. The edge intensity profile, shown in Figure 4a, was extracted from the image to determine the edge spread function (ESF). The ESF was then differentiated to obtain the line spread function (LSF), and a Fourier transform was applied to the LSF to derive the MTF. The resulting MTF graph is shown in Figure 4b, with the Nyquist frequency estimated to be ≈19.45 lp mm^−1^. Spatial resolution was calculated based on the spatial frequency at which the MTF dropped to 0.1. The corresponding spatial frequency was 13.27 lp mm^−1^ and the calculated spatial resolution was 37.68 µm. Despite the large focal spot size (400 µm), the MTF results demonstrated that its impact on spatial resolution was effectively mitigated. With the system's spatial resolution now quantified, we turned our attention to evaluating its performance in realistic imaging conditions.

### 3.2 3D Imaging of a Pouch‐Type Cell

To evaluate the high‐speed imaging performance of the developed X‐ray CL system for pouch‐type cells, 3D X‐ray imaging experiments were performed. The pouch cells have a nominal voltage of 3.7 V and a capacity of 75 Ah, with dimensions of 125 mm × 365 mm × 12 mm thickness. Since defects in pouch‐type cells frequently occur near the edge region, the inspection region was selected to examine the stacking condition of electrodes at the corners.^[^ ^43^, ^44^ ^]^ To investigate image quality under various scan times and X‐ray tube voltages, signal‐to‐noise ratio (SNR) and contrast‐to‐noise ratio (CNR) were measured from reconstructed slices.

(11) $$SNR=20\times \log\left(\frac{s}{n_{RMS}}\right)\;$$

(12) $$CNR=\frac{s-c}{\sigma_{n}}$$

where s is the mean value of an area containing cathode electrodes, *n*_RMS_ is the root mean square of a background area, *c* is the mean value of an area containing a void region in the cell. For example, an empty area without electrodes was used. Here, *σ*_n_ denotes the standard deviation of the background region.

To determine the optimal X‐ray tube voltage, projections were acquired at evenly spaced angles over 184.20° with an exposure time of 200 ms, while varying the tube voltage. ≈180° of rotation was used to avoid significant increases in X‐ray path length through non‐target regions, which can result in undesired attenuation and a subsequent reduction in SNR. An additional 4.20° was included in the scan range to ensure complete reconstruction.^[^ ^32^ ^]^ During acquisition, the X‐ray source was positioned 280 mm horizontally and 111 mm vertically from the center of the scintillator. The effective pixel size was 25.71 µm, and the field of view (FoV) was 1600 × 1600 pixels, corresponding to 41.136 mm × 41.136 mm. To minimize the effect of the missing‐wedge artifact inherent in laminography, the geometry was adjusted so that the electrode layers were aligned parallel to the z‐axis (Figure S2, Supporting Information). In this configuration, the inspection plane for electrode alignment corresponded to the *x–y* plane, effectively reducing the contribution of the missing‐wedge region in Fourier space. Under these conditions, 800 projections were obtained and subsequently used to reconstruct 3D images. The reconstruction was performed using a filtered backprojection (FBP) algorithm implemented in the CERA Xplorer software (Siemens Healthineers, Germany).

**Figure 5:**
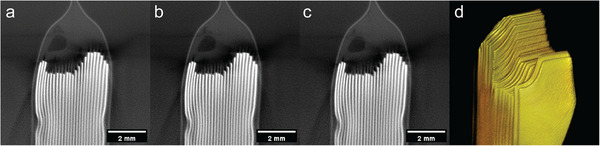
Reconstructed slices of a pouch‐type cell at different X‐ray tube voltages at voxel size = 25.71 µm, 200 ms exposure time, 800 projections, 400 W power. a) 80 kVp. b) 100 kVp. c) 120 kVp. d) 3D rendering image of (b).

**Figure** **5**a–c displays the reconstructed *x‐y* cross‐sectional image of the pouch‐type cell, clearly showing the stacked electrode structure. Since CL has reduced vertical resolution, the required cross‐section was set in the *x‐y* plane for optimal visualization.^[^ ^45^ ^]^ 3D rendering image of pouch‐type cell (Figure 5d) clearly illustrates the stacked electrode structure, including the detection of notches at the edge of the electrodes.

The reconstructed images exhibited SNRs of 37.38, 41.57, and 43.27 dB at 80, 100, and 120 kVp, respectively. The corresponding CNRs were 31.86, 35.42, and 34.72. As the X‐ray tube voltage increases, the mean energy of the polychromatic X‐ray spectrum shifts higher, resulting in greater photon penetration through the pouch‐type cell. This leads to a higher number of visible light photons generated in the scintillator, thereby increasing the overall signal and improving the SNR. However, as the mean energy increases, a larger fraction of X‐rays may transmit through the cell without sufficient attenuation, thereby reducing the contrast between materials. Consequently, although SNR improved at 120 kVp, the CNR slightly decreased compared to 100 kVp. Since CNR is considered more critical in defect inspection,^[^ ^46^ ^]^ the maximum CNR observed at 100 kVp indicates that this voltage is the most suitable for high‐speed imaging of pouch‐type cells.

**Figure 6:**
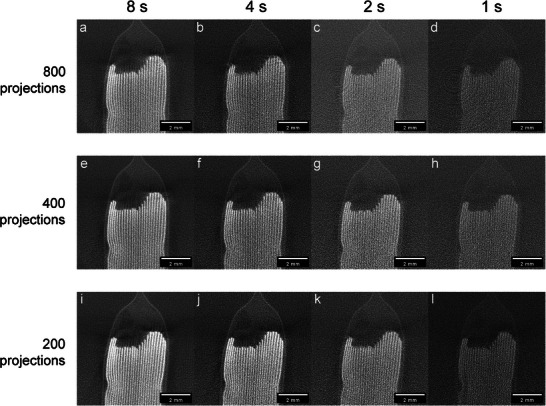
Reconstructed slices of a pouch‐type cell acquired at different scan times and numbers of projections. All scans were performed at 100 kVp and 400 W power. a–d) 800 projections; e–h) 400 projections; i–l) 200 projections. Scan times in each row are 8, 4, 2 , and 1 s from left to right.

With the tube voltage fixed at 100 kVp, high‐speed scans were performed by varying the scan time and number of projections. The other parameters remained the same as in the previous experiment. **Figure** **6** shows reconstructed slices of the pouch‐type cell obtained under different combinations of projection number and scan time. In Figure 6a–d, as the scan time decreases from 8 to 1 s at 800 projections, increased noise and more pronounced ring artifacts are observed. In Figure 6e–h, acquired with 400 projections, the exposure time per projection is doubled compared to the 800‐projection case at the same scan time, resulting in stronger signal intensities and reduced visibility of ring artifacts. In Figure 6i–l, the projection number is further reduced to 200, increasing the exposure time per projection for the same total scan time. As a result, less background noise is observed compared to the 400 and 800 projection cases. As the number of projections decreases, under‐sampling effects become more evident. In Figure 6i, vertical stripe artifacts appear due to aliasing, consistent with violations of the Nyquist sampling criterion.

**Table** **2** summarizes the corresponding SNR and CNR values under each condition. At a fixed number of projections, both SNR and CNR decrease as the scan time shortens, due to reduced photon statistics per projection and higher noise contribution. Likewise, at a fixed scan time, both SNR and CNR increase as the number of projections decreases, since the exposure time per projection becomes longer. Interestingly, at a fixed exposure time, SNR and CNR exhibit opposite trends with respect to the number of projections. As expected, the CNR decreases with fewer projections because fewer angular views lead to poorer Fourier space coverage, reducing contrast between structures. On the other hand, the SNR was observed to increase as the number of projections decreased. This counterintuitive result may be attributed to the smoother appearance of reconstructed slices with fewer projections, which reduces background noise and increases the SNR despite lower spatial fidelity. For instance, with a fixed exposure time of 10 ms per projection, reconstructions were performed using 800, 400, and 200 projections (corresponding to scan times of 8 s, 4 s, and 2 s, respectively). The mean signal intensity (s) in the source region decreased with fewer projections: 29120.88 for 800, 26118.40 for 400, and 23413.80 for 200. However, the background noise (*n*_RMS_) decreased even more significantly: 895.54, 625.16, and 512.36, respectively. Since the decrease in noise outweighed the decrease in signal, the resulting SNR increased.

**Table 2:** SNR and CNR of the pouch‐type cell as a function of scan time.

# of projections		8 s	4 s	2 s	1 s
800	SNR	30.24	28.41	26.32	13.33
	CNR	6.099	3.770	2.297	1.927
400	SNR	34.78	32.42	28.80	27.44
	CNR	6.797	5.086	3.196	1.974
200	SNR	36.77	36.46	33.20	30.54
	CNR	7.754	5.681	3.931	2.353

Industry standards further specify CNR ≥ 2.5 as a minimum benchmark for radiographic image quality in automated inspections.^[^ ^47^ ^]^ Based on this criterion, reliable inspection of pouch‐type cells is feasible at 4 s for 800 projections, and at 2 s for both 400 and 200 projections. These conditions ensure sufficient feature contrast without requiring additional post‐processing, such as averaging. Therefore, we confirm that reliable 3D imaging of pouch‐type lithium‐ion cells can be achieved within 2 s. For reference, comparative scans were also performed using a conventional micro‐CT system (ZEISS Xradia 520 Versa) under comparable voxel sampling conditions. The results, shown in Figure S3 (Supporting Information), confirm that the proposed optically‐coupled X‐ray CL system achieves comparable detectability within a significantly shorter scan time.

## 4 Conclusion

An optically‐coupled X‐ray CL system was proposed for high‐speed inspection of LIBs. Unlike conventional CT systems that rely on geometric magnification and exhibit degraded spatial resolution with increasing focal spot size, the proposed system effectively decouples spatial resolution from focal spot size by placing a scintillator in contact with the object while employing optical magnification. In the developed X‐ray imaging system, a spatial resolution of 38 µm was achieved using an X‐ray tube with a 400 µm focal spot. This novel configuration enables the use of high‐power X‐ray sources, leading to significantly reduced scan times without sacrificing image quality. High‐quality 3D X‐ray images (CNR > 3, voxel size of 25.71 µm) of a pouch‐type cell were acquired in as short as 2 s. These results demonstrate the capability of the system for rapid, high‐resolution imaging and feasibility for high‐throughput in‐line inspection in mass‐production LIB manufacturing environments. This enhanced inspection speed offers substantial advantages in manufacturing efficiency and quality assurance, potentially mitigating critical safety risks associated with internal defects in battery cells. Furthermore, the presented approach can be readily extended to various LIB formats and semiconductor devices, thus broadening its industrial applicability. We believe that this study introduces a new design strategy for advanced X‐ray imaging systems by resolving the long‐standing trade‐off between spatial resolution and scan time, thereby enabling remarkable improvements in inspection performance and productivity across diverse industrial sectors.

## Conflict of Interest

The authors declare no conflict of interest.

## Supporting information



Supporting Information

## Data Availability

The data that support the findings of this study are available from the corresponding author upon reasonable request.
