# Supplementary material for: Optically‐Coupled X‐Ray Computed Laminography System for High‐Speed Inspection of Lithium‐Ion Batteries
Source: Adv Sci (Weinh). 2025 Nov 21;13(7):e17158. doi: 10.1002/advs.202517158 (PMC12866757; doi:10.1002/advs.202517158)
Supplement: Supplementary file 1 — Supporting Information [file ADVS-13-e17158-s001.pdf]

Supporting Information

Optically-coupled X-ray Computed Laminography System for High-speed Inspection of Lithium-ion Batteries

*Jaeyoung Im, Jun Heo, Seunguk Cheon, Jichan Kim, and Sung Oh Cho**

Derivation of Equation 8:

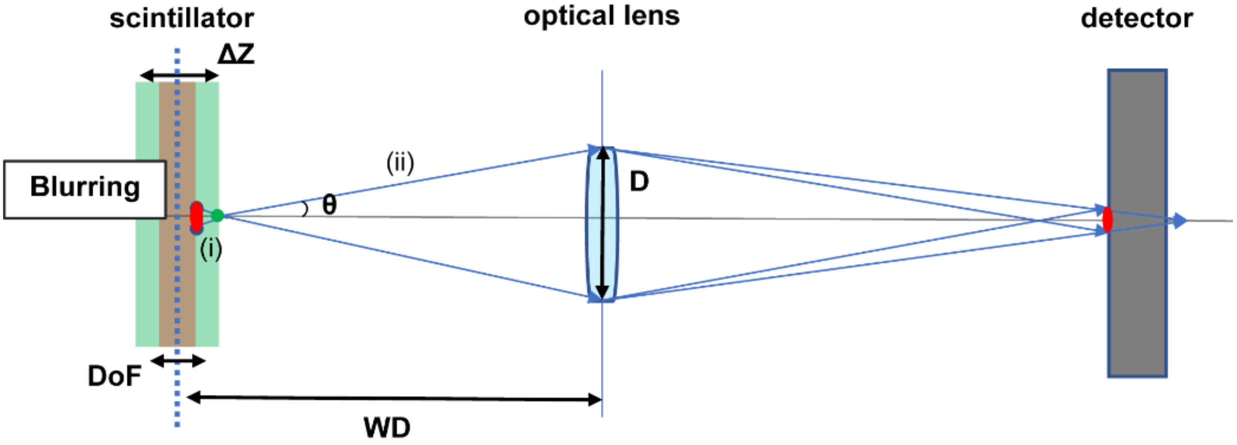

Figure S1. Schematic illustrating blurring caused by the mismatch between scintillator thickness and the DoF of optical system.

As illustrated in **Figure S1**, light emitted from regions of the scintillator that lie outside the DoF of the optical system contributes to image blurring when it enters the lens. The extent of this blurring can be estimated using geometric similarity. Assuming that the optical focal plane lies at the midpoint of the scintillator thickness (i.e., at a distance equal to the working distance (WD) from the lens), the two triangles formed in Figure S1—(i) the small triangle traced by a marginal ray within the scintillator, and (ii) the larger triangle formed between the scintillator surface and the lens aperture—are geometrically similar. Based on this similarity, the following proportion holds:

$$\frac{\Delta Z - DoF}{2} : \left(WD - \frac{\Delta Z}{2}\right) = Blurring : D \tag{S1}$$

$$Blurring = \frac{\Delta Z - DoF}{2} \times \frac{D}{WD - \frac{\Delta Z}{2}} \approx \frac{\Delta Z - DoF}{2} \times \frac{D}{WD} \tag{S2}$$

Assuming the scintillator-induced blurring follows a Gaussian PSF, the spatial resolution degradation due to DoF mismatch can be estimated as:

$$R_{scint} = \frac{Blurring}{2.355} = \frac{\Delta Z - DoF}{2 \times 2.355} \times \frac{D}{WD} \tag{S3}$$

Let θ_{scint} denote the angle at which visible photons emitted near the edge of the scintillator's DoF pass through the scintillator–air interface.

$$\sin(\theta_{scint}) = \frac{D}{2(WD - \frac{DoF}{2})} \approx \frac{D}{2\,WD} \; (\because \frac{DoF}{2} \ll WD) \tag{S4}$$

Substituting this into the Equation S3,

$$R_{scint} = \frac{\Delta Z - DoF}{2.355} \times \sin(\theta_{scint}) \tag{S5}$$

Applying Snell’s law at air-scintillator interface, we obtain:

$$n_{air} \times \sin(\theta_{air}) = n_{scint} \times \sin(\theta_{scint}) \tag{S6}$$

$$\sin(\theta_{scint}) = \frac{n_{air} \times \sin(\theta_{air})}{n_{scint}} = \frac{NA}{n_{scint}} \tag{S7}$$

Substituting this result into the spatial resolution expression yields:

$$\therefore \; R_{scint} = \frac{\Delta Z - DoF}{2.355} \times \frac{NA}{n_{scint}} \tag{S8}$$

Geometry adjustment to avoid missing-wedge artifacts:

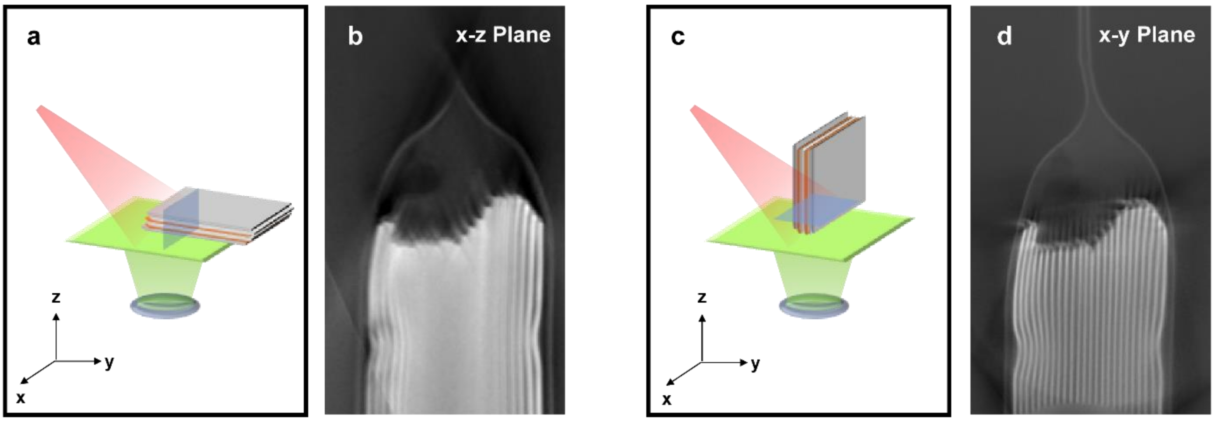

Figure S2. Geometry adjustment to avoid missing-wedge artifacts in laminography of pouch cells. a) Imaging geometry in which the inspection cross-section for electrode alignment lies in an x–z plane (semi-transparent blue plane). b) Reconstructed slice from a). c) Imaging geometry in which the inspection cross-section for electrode alignment lies in an x–y plane (semi-transparent blue plane). d) Reconstructed slice from c).

To avoid geometric distortion and artifacts caused by the missing-wedge near the z-axis in Fourier space, the imaging geometry was adjusted. When the inspection cross-section for electrode alignment includes the z-axis, as shown in Figure S2a, the reconstructed 3D image exhibits geometric distortion and streaks, which hinder reliable assessment of the electrode structure (Figure S2b). To address this, the inspection plane was set to the x–y plane (Figure S2c). This adjusted geometry effectively avoids the missing-wedge region, resulting in a reconstructed image that is free from the distortion and artifacts (Figure S2d).

Comparative micro-CT scans:

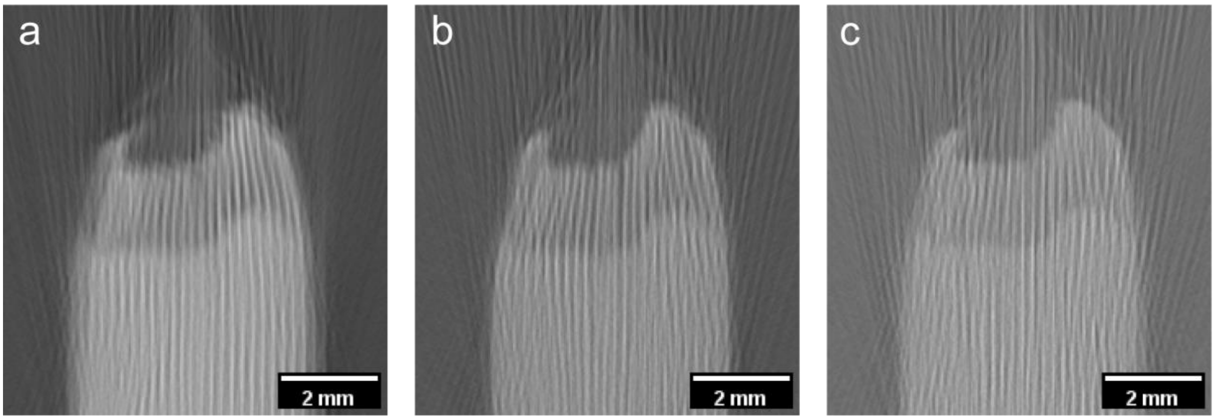

Figure S3. Reconstructed slices of a pouch-type cell acquired with a ZEISS Xradia 520 micro-CT system at different numbers of projections. All scans were performed at 100 kVp, 9 W power, 1 s exposure time per projection, and 26 μm voxel size. a) 800 projections; b) 400 projections; c) 200 projections.

For comparison with the proposed optically-coupled CL system, conventional micro-CT imaging was conducted on the same pouch cell under identical conditions. Each projection was acquired at 100 kVp and 9 W, corresponding to the maximum tube power, with an exposure time of 1 s per projection. The reconstructed images obtained from 800, 400, and 200 projections yielded CNR values of 4.334, 3.005, and 1.910, respectively. To achieve comparable image quality, the conventional micro-CT system required approximately 400 s scan time, whereas the proposed CL system achieved similar feature detectability within 2 s.
